# Coenzyme Q10 Improves the Post-Thaw Sperm Quality in Dwarf Surfclam *Mulinia lateralis*

**DOI:** 10.3390/antiox13091085

**Published:** 2024-09-04

**Authors:** Zhen Xu, Zujing Yang, Lisui Bao, Bei Lu, Xiaoxu Li, Xin Zhan, Xiaoting Huang, Yibing Liu

**Affiliations:** 1 Key Laboratory of Marine Genetics and Breeding (Ministry of Education), College of Marine Life Sciences, Ocean University of China, Qingdao 266003, China; xuzhen3196@stu.ouc.edu.cn (Z.X.); yzj@ouc.edu.cn (Z.Y.); lubei@stu.ouc.edu.cn (B.L.); 2 Institute of Evolution & Marine Biodiversity, Ocean University of China, Qingdao 266003, China; baolisui@ouc.edu.cn; 3 Fang Zongxi Center for Marine EvoDevo, Ocean University of China, Qingdao 266100, China; 4 Aquatic Sciences Centre, South Australian Research and Development Institute, Adelaide 5024, Australia; xiaoxu.li@sa.gov.au; 5 School of Marine Biology and Fisheries, Hainan University, Haikou 570228, China; 992679@hainanu.edu.cn; 6 Key Laboratory of Mariculture (Ministry of Education), Fisheries College, Ocean University of China, Qingdao 266003, China

**Keywords:** dwarf surf clam, sperm cryopreservation, coenzyme Q10, reactive oxygen species, lipid peroxidation, fatty acids, sperm structure

## Abstract

Previous studies have shown that post-thaw sperm performance is affected by multiple stressors during cryopreservation, such as those induced by physical, chemical, mechanical and physiological changes. One of these is the balance disturbance between the antioxidant defense system and reactive oxygen species (ROS) production. This study investigated whether this disturbance could be alleviated by the addition of different antioxidants to cryoprotective solution [8% dimethyl sulfoxide (DMSO) in 1 µm filtered seawater] optimized for the sperm in dwarf surf clam *Mulinia lateralis*, the model bivalve species used in many different types of studies. Results showed that the addition of 20 μM coenzyme Q10 (Q10) to 8% DMSO achieved a D-stage larval rate similar to that of the fresh control at a sperm-to-egg ratio at least 50% less than the 8% DMSO treatment alone. The addition of other antioxidants (glycine, melatonin and polyvinylpyrrolidone) did not have any positive effects. The improvement in post-thaw sperm quality by Q10 could be due to its ability to significantly decrease ROS production and lipid peroxidation and significantly increase the motility, plasma membrane integrity, mitochondrial membrane potential, acrosome integrity, DNA integrity and activities of catalase and glutatione. In this study, 37 fatty acids (FAs) were quantified in dwarf surf clam sperm, with 21 FAs being significantly impacted by the cryopreservation with 8% DMSO. Thirteen of these 21 FAs were changed due to the addition of 20 μM Q10 to 8% DMSO, with approximately half of them being improved significantly toward the levels of fresh control, while the remaining half extended further from the trends shown with 8% DMSO treatment. However, no significant difference was found in the percentage of each FA category sum and the ratio of unsaturated/saturated FAs between the two treated groups. In conclusion, the antioxidant Q10 has shown the potential to further improve the sperm cryopreservation technique in bivalves.

## 1. Introduction

The global community is experiencing an urgent challenge to provide nutritious food for the projected population of approximately 10 billion by 2050 [1]. Aquaculture is the fastest-growing food sector in the world and has been generally recognized as a critical development strategy to address this challenge [2,3,4]. Mollusks are the second largest category of farmed seafoods by quantity with the main species currently farmed all being marine bivalve species (e.g., oysters, clams, cockles, scallops and mussels) [5]. In comparison with livestock and other aquaculture species, marine bivalve species have higher nutrient compositions (per edible unit) required by humans, such as protein, omega-3, iron, zinc and vitamins [3] and smaller environmental footprints, such as low greenhouse gas emissions and limited use of land and fresh water [6]. Therefore, the further extension of this sector would represent a key opportunity for the sustainable food supply.

Genetic improvement programs with selective breeding, ploidy manipulation and hybridization techniques have been initiated in many marine bivalve species to improve production and ensure the long-term sustainable development and competitive advantage of the industry [7,8,9]. The potential for these genetic improvement programs would be further enhanced if the existing key bottlenecks could be addressed, including synchronizing spawning between males and females within and between species, extending the short window period of natural spawning and minimizing the risks of maintaining the life and health of superior broodstock [10]. Sperm cryopreservation has been widely acknowledged as an effective and reliable technique to address these bottlenecks and has been integrated into livestock breeding programs, resulting in a billion-dollar business globally [10]. Conversely, this technique has not been applied in bivalves, although it has been investigated in many species [11], especially oysters [10]. One of the main reasons is the lack of sufficient information on the long-term fitness of resultant progenies and across generations, as negative effects have been reported recently in the European eel *Anguilla anguilla* [12] and the brown trout *Salmo trutta* [13]. Another reason is low post-thaw sperm quality, resulting in high sperm-to-egg ratios (up to 160,000:1) required at fertilization [11,14].

The dwarf surf clam *Mulinia lateralis* is a dioecious marine bivalve species and has been used as a model species in both genetic [15] and cryopreservation [16] studies due to its short life cycle (about three months) and a well-established laboratory maintenance protocol [17]. In addition, a recent study by Xu et al. [16] showed that the non-programmable sperm cryopreservation technique commonly used in marine bivalve species is also suitable for dwarf surf clams. Therefore, this species could provide a unique opportunity to accelerate investigations into eliminating the bottlenecks affecting the application of sperm cryopreservation in bivalves.

Cryodamage is inevitable in sperm cryopreservation and compromises the performance of resultant progeny. Understanding the effects of cryodamage on sperm quality has been considered as a critical component to improve cryopreservation techniques in livestock [18,19] and finfish species [20]. During the cryopreservation process, sperm experience a range of stresses that could impact their quality, such as those induced by temperature shock, dehydration and mechanical (e.g., ice crystal formation) or chemical (e.g., toxicity of cryoprotectant agents) damages [10,11,19,20]. The balance breakdown between the antioxidant defense system and reactive oxygen species (ROS) production is known as oxidative stress, and it is a key factor affecting post-thaw sperm quality in livestock (e.g., cattle, horse, pig and sheep) [19] and finfish species (e.g., gilthead seabream *Sparus aurata* and common carp *Cyprinus carpio*) [21]. This stress can be effectively alleviated by antioxidant supplementation in cryoprotective solutions in cattle [19], sheep [22], horses [23], roosters [24] and gilthead seabream [21]. However, the potential benefits of antioxidants in sperm cryopreservation have not been exploited in shellfish species.

Coenzyme 10 (Q10), melatonin, polyvinylpyrrolidone (PVP) and glycine have demonstrated positive antioxidant impacts in sperm cryopreservation. For example, post-thaw sperm ROS production was significantly reduced when Q10 (2 µM), melatonin (0.1 mM) or PVP (0.5%) was added to cryoprotective solution in rooster [24], rabbit [25] and pig [26], respectively. The post-thaw sperm total antioxidant capacity was significantly increased after the addition of 10 mM glycine in cattle [27]. However, their antioxidant property and related roles in molluscan sperm cryopreservation are still not clear. In this study, their impacts on dwarf surf clams were evaluated using the post-thaw sperm performance indicator (D-stage larval rate) first. The antioxidant (Q10) that could improve the survival of resultant progeny was further investigated with a comprehensive set of sperm quality assessment parameters, including ROS production, lipid peroxidation, motility, plasma membrane integrity (PMI), mitochondrial membrane potential (MMP), acrosome integrity (AI), activities of catalase (CAT), superoxide dismutase (SOD) and glutathione (GSH), DNA integrity and changes in fatty acid (FA) profiles, to understand its antioxidant property and other cryoprotective roles.

## 2. Materials and Methods

### 2.1. Gamete Collection and Cryoprotective Solution Preparation

The mature dwarf surf clam were supplied by the Ministry of Education Key Laboratory of Marine Genetics and Breeding, Ocean University of China (Qingdao, China). The methods for gamete collection were the same as those described by Xu et al. [16]. Briefly, spawning was induced individually by thermal shock (~26 °C for about 40 min). After debris (mainly mucus and feces) was removed with a 24 µm sieve, the same volume of sperm with a motility rate > 85% was pooled from at least ten individuals in each experiment. The sperm concentration was diluted to 1 × 10^8^ cells mL^−1^ with 1 µm (nominal) filtered seawater (FS) and stored on ice prior to usage in experiments [16]. The eggs were gently poured into a 24 µm sieve with a 90 µm sieve on top to remove the debris. Eggs without signs of fertilization (no polar body or cell division) were pooled from at least five individuals in each experiment, and their density was diluted to 1 × 10^4^ cells mL^−1^ with FS [16]. Different batches of males and females were used in different experiments. Both sperm and eggs were used in the subsequent experiments within 3 h of spawning.

The cryoprotective chemical [dimethyl sulfoxide (DMSO)] and the antioxidants (glycine, melatonin, coenzyme Q10 and polyvinylpyrrolidone) were purchased from Sigma-Aldrich Pty Ltd. (St. Louis, MO, USA). The cryoprotective stock solution was prepared in FS at a concentration twice that of the final concentration required. Therefore, when the sperm were mixed with stock solution at a 1:1 ratio (*v*:*v*), the required final concentration was produced.

### 2.2. Experimental Design

In this study, each treatment was replicated three times with different batches of sperm and fertilized with the same pool of eggs. The control (fresh sperm) was also replicated three times with different batches of sperm and eggs.

#### 2.2.1. Effects of Types and Concentrations of Antioxidant on Post-Thaw D-Stage Larval Rate

In this experiment, the effect of the addition of glycine (0.3%, 0.6%, 1.2% and 2.4%), PVP (0.05%, 0.1%, 0.2% or 0.4%), Q10 (5 µM, 10 µM, 20 µM or 40 µM) or melatonin (0.5 mM, 1 mM, 2 mM or 4 mM) into 8% DMSO was assessed. The working concentrations were established according to the results from our preliminary experiments and information from published papers in other species [24,25,26,27]. The procedures for sperm cryopreservation were the same as those described by Xu et al. [16]. Briefly, the ice-cold fresh sperm (4 °C) were mixed with ice-cold cryoprotective solutions (4 °C) at a 1:1 ratio (*v*:*v*). After 10 min equilibration on ice, the sperm + cryoprotective solution mixture was transferred to 0.25 mL straws (IMV, L’Aigle, France) and placed on a rack 4 cm above the liquid nitrogen (LN) surface. The straws were then exposed to LN vapor for 10 min before being stored in LN for at least 12 h. The sperm were thawed and recovered in 60 °C (5 s) and 21 °C seawater baths (<60 s), respectively. The thawed sperm were transferred into 5 mL tubes at room temperature (~21 °C), and a subsample from post-thaw sperm was used directly for fertilization at a sperm-to-egg ratio of 1100:1. The controls (fresh sperm) were established at a sperm-to-egg ratio of 5:1 [28].

#### 2.2.2. Comparison of Sperm-to-Egg Ratios on D-Stage Larval Rate between the Sperm Cryopreserved with 8% DMSO and 8% DMSO + 20 µM Q10

As sperm cryopreserved with either 8% DMSO + 20 µM Q10 or 8% DMSO resulted in a D-stage larval rate similar to that of the fresh sperm in the previous assay, this experiment was to determine if the addition of Q10 could improve post-thaw sperm quality by assessing sperm-to-egg ratios (300:1, 500:1, 700:1 and 900:1) lower than those used in Section 2.2.1 (1100:1). The other procedures were the same as in the previous section.

#### 2.2.3. Comparison of Sperm Quality between Fresh and Cryopreserved Sperm

According to the results from the previous two experiments, the quality of sperm cryopreserved with 8% DMSO + 20 µM Q10 and 8% DMSO and the fresh sperm were compared in this experiment. A set of parameters, including ROS production, lipid peroxidation, motility, PMI, MMP, AI, enzyme activity, DNA integrity and FAs were used to assess sperm quality.

### 2.3. Sperm Quality Evaluation Methods

Sperm quality in this study was assessed in terms of motility, D-stage larval rate, ROS production, lipid peroxidation, PMI, MMP, AI, activities of CAT, SOD and GSH, DNA integrity and changes in FA profiles.

Sperm motility was assessed by diluting sperm suspension to 1 × 10^7^ cells mL^−1^ and expressed as the percentage of active sperm out of 100 under a microscope at 200× magnification [29,30]. Sperm moving forward progressively were counted as active sperm, while those vibrating or not moving at all were counted as dead sperm [29,30]. Each sample was assessed by two independent observers.

For D-stage larval rate assessment, 1 mL concentrated eggs (10,000 eggs mL^−1^) were taken and mixed gently with sperm to reach the predetermined sperm-to-egg ratio in the experiments. After mixing with sperm for 15 min, the eggs were washed gently on a 24 µm sieve with FS before being cultured in a 500 mL container at 21 °C. The D-stage larval rate was determined 24 h post-fertilization and calculated as the percentage of eggs that developed into D-stage larvae [16].

Intracellular ROS production was determined by a commercial fluorometric assay kit (MAK143, Sigma-Aldrich, USA) according to the manufacturer’s instructions. The 1 mL sperm samples (2 × 10^7^ cells mL^−1^) were centrifuged at 72× *g* for 2 min. The sperm pellets were resuspended in ice-cold (4 °C) PBS (500 µL) and transferred to cell plate. A 100 μL quantity of Master Reaction Mix (provided by the kit) was then added and incubated at 37 °C for 1 h. The fluorescence intensity was measured using the microplate reader (Infinite 200 PRO, Tecan, Grödig, Austria). Results were expressed in relative fluorescence unities (RFU) at λex = 490 nm/λem = 525 nm.

The lipid peroxidation was detected using the assay kit (ab118970, Abcam, Cambridge, UK) according to the procedures detailed by Xu et al. [16]. Briefly, 1 mL sperm (2.5 × 10^7^ cells mL^−1^) was homogenized using 300 µL malondialdehyde (MDA) lysis buffer and centrifuged at 13,000× *g* for 10 min at 4 °C to remove the insoluble material. Then 200 μL supernatant was incubated with 600 μL thiobarbituric acid for 60 min at 95 °C. After being cooled to room temperature, 300 μL n-butanol and 100 μL sodium chloride (5 M) were added to the sample and mixed with a vortex mixer. Subsequently, the samples were centrifuged at 16,000× *g* for 3 min at room temperature. The supernatant was collected and heated to 55 °C to evaporate the n-butanol. The residue was resuspended in 200 µL ddH_2_O before being used to measure the absorbance of MDA at 532 nm with the microplate reader.

The fluorescent agents were purchased from Invitrogen, Shanghai, China [LIVE/DEAD sperm viability kit (L-7011) and LysoTracker (Eugene, OR, USA) green DND-26 (LYSO-G) kit (L-7526)] and Sigma-Aldrich Pty Ltd. [Rhodamine 123 (Rh123) and propidium iodide (PI)]. The sperm PMI, MMP and AI were evaluated by the SYBR14/PI (both from L-7011 kit), Rh123/PI and LYSO-G (from L-7526 kit)/PI methods, respectively [16]. The post-thaw sperm were diluted to 2 × 10^6^ sperm mL^−1^, and 1 mL diluted sperm was then stained. For PMI and MMP evaluations, 100 µL SYBR14 (4 µM) or Rh123 (10 µM) was added for 20 min and then 100 µL PI (200 µM for PMI and 130 µM for MMP) for a further 10 min. For AI evaluation, 5 µL LYSO-G (1 mM) was added for 30 min, and then 9 µL PI (3000 µM) for a further 10 min. The staining was carried out at room temperature, and the samples were analyzed by flow cytometry (Beckman, CytoFLEX SRT, Indianapolis, IN, USA) with a 488 nm argon-ion laser. The 525/40 nm fluorescent channel was used to detect green fluorescence, and the 585/42 nm fluorescent channel was used to detect red fluorescence. At least 20,000 events were collected from each sample. The data were analyzed using CytExpert SRT software (v1.1.0.10007).

The activity of CAT, SOD or GSH was measured using the methods detailed by Xu et al. [16]. Briefly, 1 mL sperm samples (2.5 × 10^7^ cells mL^−1^) were centrifuged at 250× *g* for 10 min at 4 °C to obtain sperm pellets first. (1) For the SOD activity assessment, the pellets were resuspended in 750 µL ice-cold PBS before being sonicated for 1 min. The supernatant from each sample was transferred into another microcentrifuge tube after being centrifuged at 1500× *g* for 10 min at 4 °C. The level of SOD activity was then determined by the EIASODC kit (Thermo Fisher Scientific, USA). The SOD concentration (U/mL) at 450 nm was measured using a microplate reader. The final values were calculated from standard curves. (2) For CAT activity analysis, the pellets were resuspended in 1 mL ice-cold 1 × assay buffer before being sonicated for 1 min. After being centrifuged at 10,000× *g* for 15 min at 4 °C, the supernatant from each sample was collected. The level of CAT activity was then determined by the EIACATC kit (Thermo Fisher Scientific, USA). The concentration (U/mL) of CAT was measured at 560 nm using the microplate reader. (3) For GSH activity assessment, the pellets were homogenized with 5% aqueous 5-sulfosalicylic acid (S2130, Sigma-Aldrich) in 250 µL ice-cold PBS and incubated for 10 min at 4 °C. The homogenized samples were then centrifuged at 18,213× *g* for 10 min at 4 °C to collect the supernatant for analysis. GSH activity (μM) was measured with the EIAGSHC kit (Thermo Fisher Scientific, USA) at 405 nm using a microplate reader.

Sperm DNA integrity was assessed by the Oxiselect comet assay kit (STA-351, Cell Biolabs, Inc., San Diego, CA, USA) with slight modifications. The sperm samples (8 × 10^5^ cells) were centrifuged (4 °C) at 700× *g* for 2 min to collect sperm pellets. The pellets were then resuspended in 100 μL ice-cold (4 °C) PBS. A 75 μL quantity of liquefied agarose was loaded onto the comet slide (provided by the kit) at 4 °C for 15 min to form a base layer. The sperm samples were mixed with molten agarose at 1:10 (*v*:*v*), and 75 μL of the mixture was loaded onto the slide at 4 °C for another 15 min. Subsequently, the slides were transferred to ice-cold lysis buffer (provided in the kit) for 3 h at 4 °C in the dark before the slides were immersed in ice-cold alkaline solution (provided in the kit) for 30 min. The slides were then moved to a horizontal electrophoresis chamber for electrophoresis in alkaline solution for 30 min (15 V and 300 mA). The samples were rinsed with ice-cold ultrapure water 2 times at a 2 min interval, placed in cold 70% ethanol (Catalog No. 10009218, Sinopharm Chemical Reagent Co., Ltd., Shanghai, China) for 5 min, dried at 37 °C for 30 min and stained with 100 μL Vista Green DNA dye (provided in the kit) for 15 min. The slides were observed using epifluorescence microscopy (DMi8, Leica, Wetzlar, Germany) with a FITC filter. The slides were analyzed by CASPLAB1.2.3 software with 100 sperm being randomly selected each time. The comet rate, damage coefficient and the five grades of DNA damage were calculated as described by Xu et al. [31]. The tail DNA content, tail length, comet length, tail moment and olive tail moment were calculated as described by Martins and Costa [32] and Shaliutina-Loginova and Loginov [33].

Sperm FAs were extracted by a method modified from Folch et al. [34]. Specifically, 200 µL sperm suspension (1.5 × 10^8^ cells mL^−1^) was mixed with ice-cold (4 °C) methanol (500 µL) and chloroform (1 mL) and ground for 5 min using a high-throughput tissue grinder (SCIENTZ-48, Ningbo, China). The mixture was then stirred vigorously for 1 min and left undisturbed at 4 °C for 30 min before being centrifuged (4 °C) at 16,260× *g* for 10 min. The organic layers were collected, transferred into a new centrifuge tube and dried with a vacuum concentrator centrifuge (ZLS-2, Changsha, China). The samples were then resuspended in 100 µL methanol/isopropanol (1:1, *v*/*v*) and sonicated before being centrifuged (4 °C) at 16,260× *g* for 15 min. The supernatant was collected to analyze the FA by a mass spectrometer (AB SCIEX 5500, GenTech scientific, Arcade, NY, USA) equipped with an ACQUITY UPLC BEH C18 column (1.7 µm film thickness, 2.1 mm × 100 mm) and liquid chromatographer (HPLC, Waters 2695, Milford, MA, USA). A 5 mM ammonium formate with mobile phase A consisting of water–acetonitrile (35:65; *v*/*v*) and phase B consisting of isopropanol–acetonitrile (65:35; *v*/*v*) were used at a flow rate of 0.3 mL/min. The samples were injected (10 µL size) and run for 10 min at 40 °C. The FAs were identified according to their retention time compared to those of standards analyzed under the same conditions [35]. The FAs were expressed as a percentage of the total FAs detected [36].

### 2.4. Statistical Analysis

Results in this study are presented as mean ± standard deviation (SD). The original data in percentage were arcsine-transformed before analysis with SPSS 22. Two-way analysis of variance (ANOVA) was applied to compare the post-thaw D-stage larval rate at different sperm-to-egg ratios. One-way ANOVA was applied to other experiments. The least significant difference (LSD) comparison was used when a significant difference was observed. Differences were considered statistically significant at *p <* 0.05.

## 3. Results

### 3.1. Effects of Types and Concentrations of Antioxidant on Post-Thaw D-Stage Larval Rate

**Figure 1:**
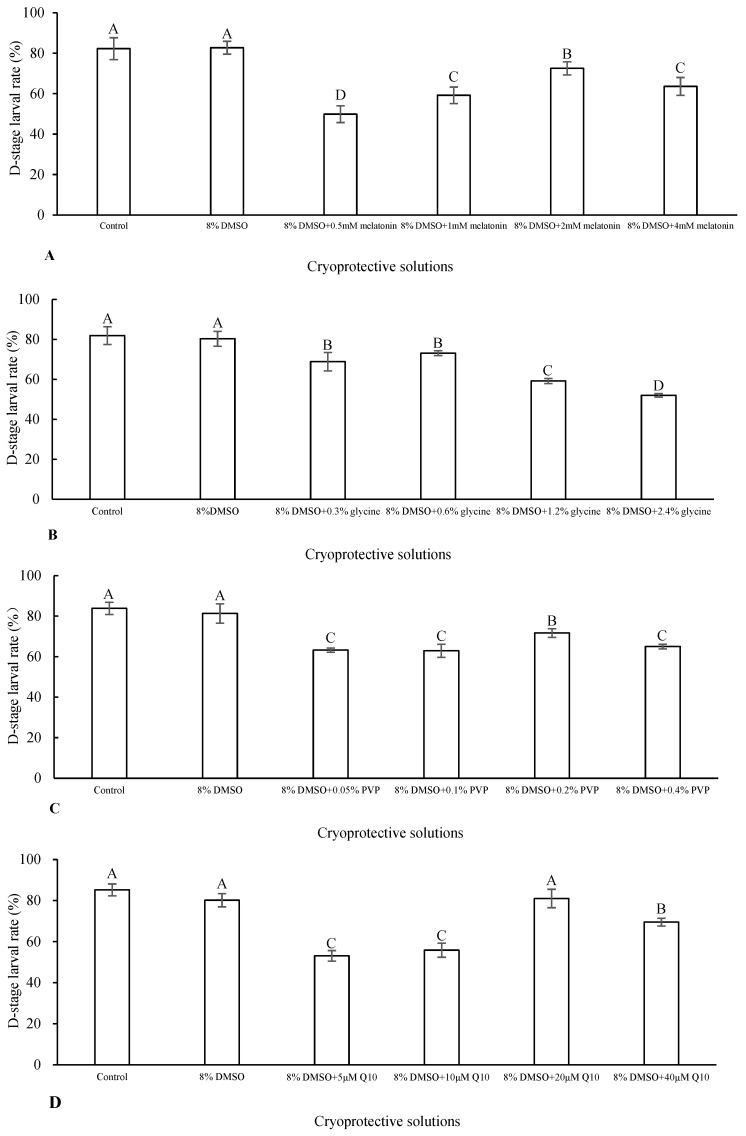
Comparison of D-stage larval rate (%) produced by fresh sperm and those cryopreserved with either 8% DMSO or its combination with an antioxidant at different concentrations, *n* = 3. (**A**–**D**) represent the addition of melatonin, glycine, PVP and Q10 into 8% DMSO, respectively. Different letters indicate significant difference (*p* < 0.05) in each antioxidant.

The D-stage larval rate was significantly decreased when the sperm were cryopreserved by adding melatonin, glycine or PVP at each of the concentrations assessed in this study (*p* < 0.05; Figure 1A–C). On the other hand, the addition of 20 µM Q10 achieved a D-stage larval rate (81.00 ± 4.50%) similar to that of the control (fresh sperm) (85.20 ± 2.89%; *p* > 0.05; Figure 1D).

### 3.2. Comparison of Sperm-to-Egg Ratio on D-Stage Larval Rate between the Sperm Cryopreserved with 8% DMSO and 8% DMSO + 20 µM Q10

**Figure 2:**
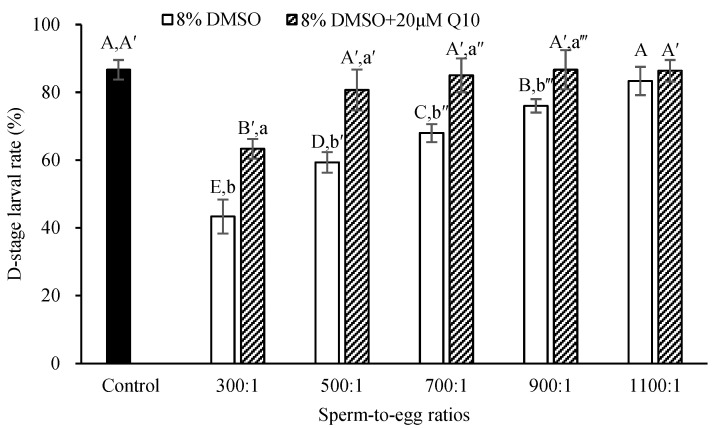
Comparison of D-larval rate (%) produced by the fresh sperm and those cryopreserved with either 8% DMSO or its combination with 20 µM Q10 at various sperm-to-egg ratios, *n* = 3. Bars with different uppercase letters within each cryoprotective solution differ significantly between sperm-to-egg ratios (*p* < 0.05). Bars with different lowercase letters within each ratio differ significantly between cryoprotective solutions (*p* < 0.05).

The D-stage larval rate increased with the increase in sperm-to-egg ratio (Figure 2). The D-stage larval rate reached a level similar to that of the control (fresh sperm) at a sperm-to-egg ratio of 1100:1 (*p* > 0.05; Figure 2) when sperm were cryopreserved with 8% DMSO or at a much lower ratio (500:1) when 20 µM Q10 was added (*p* > 0.05; Figure 2).

### 3.3. Comparison of Sperm Quality between Fresh and Cryopreserved Sperm

**Figure 3:**
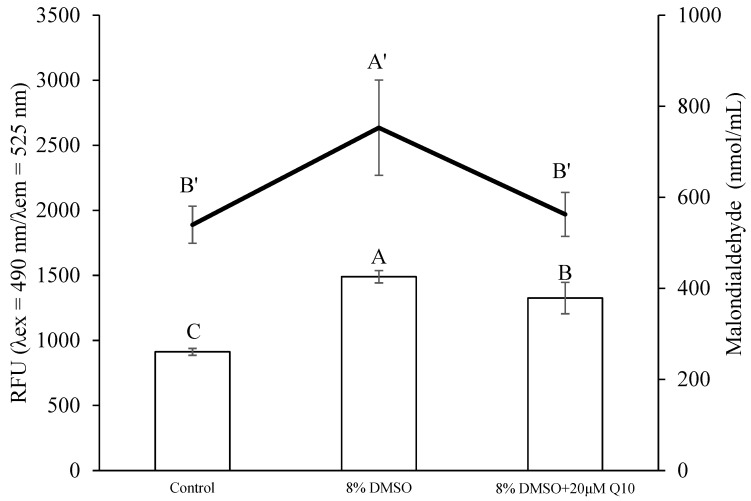
Comparison of ROS production and lipid peroxidation between the control (fresh sperm) and the sperm cryopreserved with either 8% DMSO or its combination with 20 μM Q10, *n* = 3. Different letters indicate significant difference (*p* < 0.05).

The production of ROS and the level of lipid peroxidation were both significantly increased when the sperm were cryopreserved with 8% DMSO (*p* < 0.05; Figure 3). However, both were significantly decreased by the addition of 20 µM Q10 into 8% DMSO (*p* < 0.05; Figure 3), reaching a level similar to that of the fresh control in ROS.

**Figure 4:**
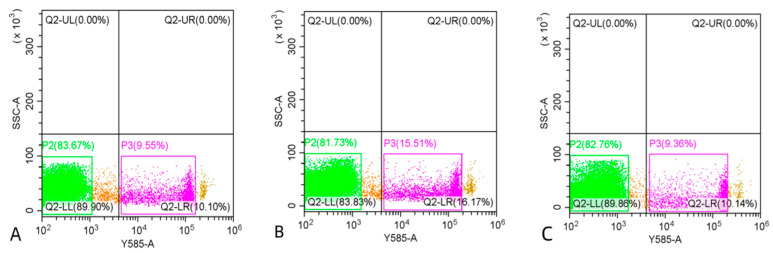
Sperm plasma membrane integrity (**A**), mitochondrial membrane potential (**B**) and acrosome integrity (**C**) analyzed by flow cytometry showing intact component/organelle (green), compromised component/organelle (pink) and debris (orange-brown).

**Figure 5:**
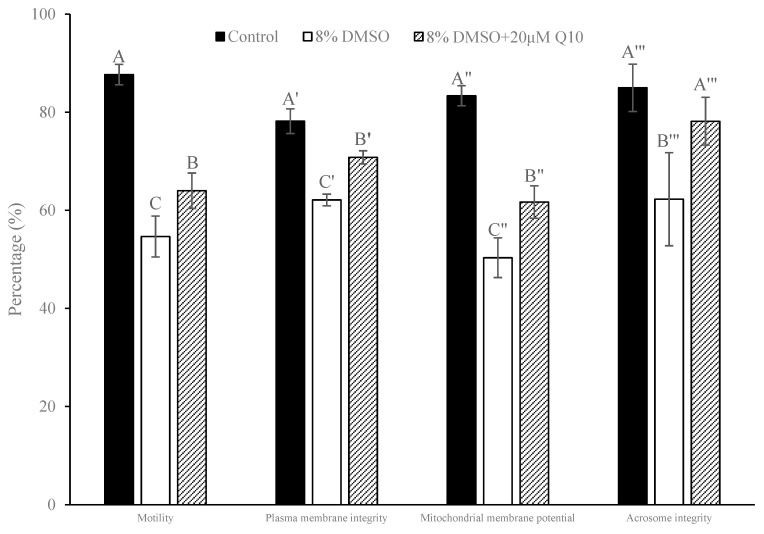
Comparison of motility, plasma membrane integrity, mitochondrial membrane potential and acrosome integrity between the control (fresh sperm) and the sperm cryopreserved with either 8% DMSO or its combination with 20 μM Q10, *n* = 3. Different letters within each parameter indicate significant difference (*p* < 0.05).

Figure 4 shows the results of sperm PMI, MMP and AI analyzed by flow cytometry. Although the percentages of motility, PMI and MMP in post-thaw sperm were significantly lower than those in the fresh control (*p* < 0.05; Figure 5), the addition of 20 µM Q10 into 8% DMSO significantly improved these parameters (*p* < 0.05; Figure 5), with the percentage of AI being increased to the level similar to the control (Figure 5).

The enzymes’ activities were significantly reduced when the sperm were cryopreserved with 8% DMSO in comparison with the fresh control (*p* < 0.05; Table 1). However, when they were cryopreserved with 8% DMSO + 20 µM Q10, the activities of CAT and GSH were significantly increased to a level similar to that of the control (*p* < 0.05; Table 1).

The classification of sperm alkaline comet assay gel electrophoresis in dwarf surf clam is shown in Supplementary Figure S1. All the parameters used to assess DNA integrity were significantly altered in the sperm cryopreserved with 8% DMSO in comparison with the fresh control (*p* < 0.05), except for grade IV (Table 2 and Table 3). The addition of 20µM Q10 to 8% DMSO significantly improved the post-thaw sperm DNA integrity (*p* < 0.05), except for the tail length, although these parameters (except for Grade I) did not return to the levels in the control (Table 2).

In total, 37 FAs were quantified in dwarf surf clam sperm with the numbers of saturated fatty acids (SFAs), monounsaturated fatty acids (MUFAs) and polyunsaturated fatty acids (PUFAs) being 17, 9 and 11, respectively (Table 4). SFAs consisted of 80.73% of the total FAs, with tridecylic acid (C13:0; 26.57%) being predominant, followed by octadecanoic acid (C18:0; 15.74%; Table 4). MUFAs made up 12.46% of the total FAs with pentadecenoic acid (C15:1; 8.23%; Table 4) being the most abundant. PUFAs were the lowest of the three categories, comprising only 6.82% of the total FAs in dwarf surf clam sperm. Of these, more than half consisted of eicosapentaenoic acid (C20:5 3.73%), followed by linoleic acid (C18:2; 1.83%; Table 4).

After sperm cryopreservation with 8% DMSO, 21 FAs were impacted, with 14 being increased significantly (4 SFAs, 6 MUFAs and 4 PUFAs) and 7 decreased significantly (4 SFAs, 1 MUFA and 2 PUFA; Table 4; *p* < 0.05). When 20 μM Q10 was added to 8% DMSO, the FAs (5 SFAs, 3 MUFAs and 5 PUFAs) that were significantly altered were all from those sensitive to cryopreservation in 8% DMSO. Seven of these FAs (3 SFAs, 2 MUFAs and 2 PUFAs) increased or decreased significantly relative to the percentage levels in the fresh sperm, whereas the remaining six (2 SFAs, 1 MUFA and 3 PUFAs) extended the trend revealed in the cryopreservation with 8% DSMO (*p* < 0.05; Table 4). 

The addition of 20 μM Q10 into 8% DMSO, however, did not alter the percentage sums of SFAs, MUFAs or PUFAs, nor the ratio of unsaturated/saturated FAs in comparison with those cryopreserved with 8% DMSO (*p* > 0.05; Table 4).

## 4. Discussion

This study shows that of the four antioxidants tested, only Q10 has the ability to further improve the non-programmable sperm cryopreservation technique by adding 20 μM to the 8% DMSO cryoprotective solution that was previously optimized for dwarf surf clams, and it results in a D-stage larval rate similar to that of fresh sperm at a sperm-to-egg ratio at least 50% lower than the ratio required in the 8% DMSO treatment. This achievement was due to the improvement in almost all the sperm quality parameters evaluated in this study. To the best of our knowledge, this is the first study to assess the effects of Q10 on sperm cryopreservation in shellfish species.

During cryopreservation, chemical damages (including ROS) are one of the primary factors compromising sperm quality [21,22]. The other key categories identified so far are physical and mechanical damages [18,19,20,37]. The addition of antioxidants to cryoprotective solution has been well acknowledged as a reliable strategy to improve post-thaw sperm quality [18,21]. The antioxidants assessed in this study have shown their capability to reduce post-thaw sperm ROS production and lipid peroxidation and/or improve the motility, fertilization, enzyme activity, mitochondrial function and integrities of DNA, plasma membrane and acrosome in other species, such as glycine in abalone *Haliotis laevigata* [38] and the monkey *Macaca fascicularis* [39]; melatonin in cattle, sheep and horses [40]; Q10 in roosters [24] and giant grouper *Epinephelus lanceolatus* [41]; and PVP in cattle [42] and Indian red jungle fowl *Gallus gallus murghi* [43]. However, only the addition of Q10 could improve the survival of resultant progeny in this study. Furthermore, at the Q10 concentrations assessed, only 20 μM positively affected post-thaw sperm quality in dwarf surf clams. Our results from Q10 agreed with the findings from sheep [44], cattle [45] and roosters [24], where the integrity of the plasma membrane and acrosome in post-thaw sperm were significantly improved by its addition. The positive role of Q10 in the improvement of post-thaw sperm CAT activity has also been shown in cattle [45], whereas the positive effect found for GSH in dwarf surf clam has not been reported in other species. In this study, the addition of Q10 did not enhance post-thaw sperm SOD activity, although this activity was improved in cattle [45]. It is widely accepted that the effects of ROS are dose-dependent [46]. For sperm to play their fundamental role, it is critical that ROS be maintained below a certain level; otherwise oxidative stress would be exerted, resulting in the malfunction of sperm [46]. Although the sperm have their own antioxidant scavenging system to ameliorate over-produced ROS to certain extent, the imbalance between ROS production and antioxidant scavenging during cryopreservation have caused cryodamage in sheep [22], cattle [18,19], pig [47], gilthead seabream [21] and giant grouper [41]. In this study, ROS production in post-thaw sperm was significantly reduced when 20 μM Q10 was added to 8% DMSO, which would be the reason for the significant decrease or increase in post-thaw sperm lipid peroxidation and DNA integrity, respectively. Similar results have also been shown in horses [23], bucks [48], roosters [24] and giant grouper [41], where the addition of Q10 could significantly decrease ROS production and lipid peroxidation and at the same time increase DNA integrity in post-thaw sperm. In a review, Kaltsas [49] pointed out that it was ubiquinol, a reduced form of Q10, that acted to protect sperm structure integrity and neutralize excessive oxidative stress during cryopreservation. 

Motility is another important indicator to evaluate sperm quality in marine bivalve species. It is correlated with mitochondrial function, such as mitochondrial respiration and ATP generation [50]. In the current study, the supplementation of 20 μM Q10 significantly improved post-thaw sperm motility and mitochondrial function. This increased ATP synthesis might be due to the mediation of Q10 bioenergetic function on the transport of protons and electrons in the mitochondrial electron transport chain [23]. Similar results have also been reported in sheep [44], cattle [45], horses [23] and giant grouper [41], with post-thaw sperm motility and mitochondrial membrane potential being significantly enhanced when Q10 was included in the cryoprotective solution. 

Lipids are one of the major constituents of sperm membrane [51]. FAs are a part of the phospholipids which contribute to sperm responses and functions, such as metabolism and membrane fluidity [52,53]. Changes to the FA profiles of sperm have been used to understand cryodamage [54,55]. In the current study, 37 FAs were identified in dwarf surf clam sperm, with SFA being the most abundant category, followed by MUFA and PUFA. A similar FA category composition has been reported in the sperm of livestock species, such as cattle [54,56] and horses [57]. However, a different category of composition has been found in fish species, such as Atlantic salmon *S. salar* [51] and gilthead seabream [58], with PUFA being the most abundant, followed by SFA and MUFA.

Normally, phospholipids with a high proportion of PUFAs increase membrane flexibility and fluidity, which are positively correlated with cryo-resistance in sperm [51,52,54]. However, a high PUFA content in the plasma membrane could also render sperm susceptible to oxidative stress [59]. It was found in this study that the sum percentage of PUFA was lower than 8% in dwarf surf clam sperm, concurring with the levels reported in cattle [56], felines [60] and some bat species [61]. However, these findings differed from the results in gilthead seabream [58], Atlantic salmon [51], common carp [62], cattle [54,63] and some marsupial species [64], where the proportion of PUFAs was high (36%~65%) with docosapentaenoic acid (C22:5n-3) and/or docosahexaenoic acid (C22:6n-3) being dominant. These two PUFAs have also shown positive correlations with sperm cryotolerance in horses [57] and pigs [65]. The percentages of these two acids were, on the other hand, extremely low in dwarf surf clam sperm (<0.2%) and that of felines [60] and some bat species [61]. Furthermore, neither were sensitive to the addition of 20 μM Q10 in this study, although docosahexaenoic acid increased after cryopreservation with 8% DMSO.

Published results show that PUFAs are vulnerable to highly reactive and short-lived ROS during cryopreservation, as ROS attacks their double bonds [54] and thus are reduced after cryopreservation [51,58,65]. The results of the current study suggested that FAs in each of the three categories (SFAs, MUFAs and PUFAs) respond differently to sperm cryopreservation with 8% DMSO in dwarf surf clam, with approximately half of the cryo-sensitive FAs being increased or decreased in each category. The causal factor for these changes is unclear, although they might be due to increased desaturase activity as low temperatures could activate the expression of desaturase genes [66]. Increases in the percentages of tetradecenoic acid (C14:1), palmitoleic acid (C16:1) and erucic acid (C22:1) have also been shown in post-thaw trochophore larvae of the mussel species *Mytilus trossulus* [66] and oleic acid (C18:1) in post-thaw sperm in pigs [65]. In this study, although the percentage of docosahexaenoic acid (C22:6) was significantly increased in post-thaw sperm, an opposite trend has been reported in other species, such as pigs [65] and Atlantic salmon [51]. Furthermore, although the addition of 20 μM Q10 significantly reduced lipid peroxidation and ROS production and maintained the total in each unsaturated category, only approximately half of those FAs affected in each category returned to the levels in fresh sperm while the remaining half further extended the trends (increase or decrease) in the sperm cryopreserved with DMSO. The reasons for these phenomena were not clear, although they could partially be explained by the defense functions of antioxidant systems in sperm previously published: (A) the prevention of free radical formation, (B) the prevention and restriction of chain reaction propagation and (C) the management of damaged molecules [67,68]. Q10 is a fat-soluble antioxidant and has a strong antioxidant capability [69]. However, the cryoprotective mechanism of Q10 needs to be further investigated to reveal if and which sperm FAs contribute to the improvements of sperm quality in bivalves.

## 5. Conclusions

This study demonstrated that the addition of antioxidant coenzyme Q10 (20 μM) to a cryoprotective agent (8% DMSO) previously optimized for dwarf surf clam sperm significantly improved all the post-thaw sperm quality parameters assessed (except for SOD), resulting in a D-stage larval rate similar to that of fresh sperm at a sperm-to-egg ratio at least 50% lower than the sperm cryopreserved with 8% DMSO. This antioxidant agent has decreased ROS production and lipid peroxidation and at the same time increased post-thaw sperm motility, the activities of catalase and glutathione, plasma membrane integrity, mitochondrial membrane potential, acrosome integrity and DNA integrity. Of the 21 FAs impacted by the cryopreservation with 8% DMSO, approximately half of them were improved significantly due to the addition of 20 μM Q10, while the remaining half further extended the trends shown with the 8% DMSO treatment. However, the addition of 20 μM Q10 to 8% DMSO did not alter the percentage sum of each FA category and the ratio of unsaturated/saturated FAs.

## Figures and Tables

**Table 1:** Comparison of the activity of SOD, CAT or GSH between the control (fresh sperm) and the sperm cryopreserved with either 8% DMSO or its combination with 20 μM Q10 *.

Enzyme Activity	Group			*p* Value
	Control	8% DMSO	8% DMSO + 20 μM Q10	
SOD (U/mL)	18.1 ± 1.0 ^A^	8.8 ± 1.5 ^B^	10.0 ± 0.5 ^B^	<0.001
CAT (U/mL)	8.5 ± 0.8 ^A′^	4.8 ± 0.7 ^B′^	7.7 ± 1.6 ^A′^	0.015
GSH (μM)	3.5 ± 0.4 ^A″^	1.7 ± 0.2 ^B″^	2.7 ± 0.7 ^A″^	0.012

* Different letters within each enzyme indicate significant difference (*p* < 0.05; *n* = 3).

**Table 2:** Comparison of different grades of DNA damage and comet rate between the control (fresh sperm) and the sperm cryopreserved with either 8% DMSO or its combination with 20 μM Q10 *.

	Group			*p* Value
	Control	8% DMSO	8% DMSO + 20 μM Q10	
Grade 0 (%)	67.7 ± 2.1 ^A^	29.7 ± 5.5 ^C^	44.3 ± 3.2 ^B^	<0.001
Grade I (%)	29.7 ± 1.5 ^B^	38.0 ± 1.00 ^A^	33.0 ± 2.6 ^B^	0.005
Grade II (%)	2.7 ± 1.2 ^C^	23.0 ± 2.6 ^A^	18.0 ± 1.0 ^B^	<0.001
Grade III (%)	0 ^C^	9.3 ± 1.2 ^A^	4.7 ± 1.2 ^B^	<0.001
Grade IV (%)	0	0	0	
Comet rate (%)	32.3 ± 2.1 ^C^	70.3 ± 5.5 ^A^	55.7 ± 3.2 ^B^	<0.001

* Different letters within each column indicate significant difference (*p* < 0.05; *n* = 3).

**Table 3:** Comparison of damage coefficient, tail DNA, tail length, comet length, tail moment and olive tail moment between the control (fresh sperm) and the sperm cryopreserved with either 8% DMSO or its combination with 20 μM Q10 *.

	Group			*p* Value
	Control	8% DMSO	8% DMSO + 20 μM Q10	
Damage coefficient	35.0 ± 3.0 ^C^	112.0 ± 15.1 ^A^	83.0 ± 4.4 ^B^	<0.001
Tail DNA (%)	5.0 ± 0.4 ^C^	16.2 ± 2.8 ^A^	11.7 ± 0.6 ^B^	<0.001
Tail length (μm)	5.5 ± 0.4 ^B^	10.5 ± 2.4 ^A^	9.1 ± 0.7 ^A^	0.014
Comet length (μm)	36.5 ± 1.6 ^C^	48.4 ± 3.2 ^A^	40.4 ± 0.2 ^B^	0.001
Tail moment	0.4 ± 0.1 ^C^	2.7 ± 0.3 ^A^	1.9 ± 0.2 ^B^	<0.001
Olive tail moment	0.7 ± 0.1 ^C^	3.0 ± 0.2 ^A^	2.0 ± 0.2 ^B^	<0.001

* Different letters within each column indicate significant difference (*p* < 0.05; *n* = 3). Damage coefficient = [(Grade 0 cell number × 0) + (Grade I cell number × 1) + (Grade II cell number × 2) + (Grade III cell number × 3) + (Grade IV cell number × 4)] [31].

**Table 4:** Comparison of fatty acid percentages between the control (fresh sperm) and the sperm cryopreserved with either 8% DMSO or its combination with 20 μM Q10 *.

Fatty Acid (%)	Control	8% DMSO	8% DMSO + 20 μM Q10	*p* Value
Saturated	80.728 ± 1.106 ^A^	76.077 ± 1.314 ^B^	77.042 ± 0.770 ^B^	0.004
Butyric acid, C4:0	5.211 ± 0.585 ^A^	2.339 ± 0.417 ^B^	1.988 ± 0.377 ^B^	<0.001
Hexanoic acid, C6:0	4.350 ± 0.942	4.705 ± 0.388	3.789 ± 0.381	0.261
Octanoic acid, C8:0	0.563 ± 0.110 ^C^	3.604 ± 0.512 ^B^	4.625 ± 0.478 ^A^	<0.001
Decanoic acid, C10:0	2.819 ± 0.659	3.203 ± 0.155	3.192 ± 0.376	0.493
Undecanoic acid, C11:0	7.222 ± 0.779	7.241 ± 0.116	7.437 ± 0.439	0.854
Dodecanoic acid, C12:0	7.107 ± 0.813	6.806 ± 0.097	7.153 ± 0.279	0.676
Tridecylic acid, C13:0	26.570 ± 2.189	24.380 ± 1.949	24.030 ± 1.003	0.252
Tetradecanoic acid, C14:0	4.447 ± 0.649 ^A^	2.792 ± 0.072 ^B^	3.397 ± 0.169 ^B^	0.004
Pentadecanoic acid, C15:0	2.038 ± 0.488	1.606 ± 0.089	1.725 ± 0.111	0.255
Hexadecanoic acid, C16:0	2.231 ± 0.110 ^A^	2.227 ± 0.125 ^A^	1.767 ± 0.065 ^B^	0.002
Heptadecanoic acid, C17:0	1.521 ± 0.182	1.740 ± 0.138	1.508 ± 0.103	0.176
Octadecanoic acid, C18:0	15.739 ± 0.503	14.760 ± 1.079	15.479 ± 1.535	0.574
Eicosanic acid, C20:0	0.808 ± 0.137 ^A^	0.381 ± 0.016 ^B^	0.775 ± 0.045 ^A^	0.001
Heneicosanoic acid, C21:0	0.050 ± 0.006 ^A^	0.013 ± 0.004 ^C^	0.025 ± 0.003 ^B^	<0.001
Docosanoic acid, C22:0	0.025 ± 0.002 ^B^	0.128 ± 0.003 ^A^	0.015 ± 0.002 ^C^	<0.001
Tricosylic acid, C23:0	0.018 ± 0.004 ^B^	0.036 ± 0.001 ^A^	0.029 ± 0.004 ^A^	0.002
Tetracosanoic acid, C24:0	0.010 ± 0.003 ^B^	0.117 ± 0.008 ^A^	0.106 ± 0.004 ^A^	<0.001
Monounsaturated	12.457 ± 1.026 ^B^	17.201 ± 1.111 ^A^	15.863 ± 0.522 ^A^	0.002
Tetradecenoic acid, C14:1	0.027 ± 0.005 ^B^	0.039 ± 0.001 ^A^	0.045 ± 0.003 ^A^	0.004
Pentadecenoic acid, C15:1	8.230 ± 0.781 ^B^	11.390 ± 0.850 ^A^	9.809 ± 0.891 ^AB^	0.011
Palmitoleic acid, C16:1	0.106 ± 0.010 ^B^	0.148 ± 0.003 ^A^	0.152 ± 0.009 ^A^	0.001
Heptadecenoic acid, C17:1	1.052 ± 0.032 ^A^	0.939 ± 0.048 ^B^	1.028 ± 0.032 ^A^	0.026
Octadecenoic acid, C18:1T	1.735 ± 0.218 ^B^	2.382 ± 0.187 ^A^	2.057 ± 0.285 ^AB^	0.041
Oleic acid, C18:1	0.203 ± 0.035	0.240 ± 0.040	0.216 ± 0.027	0.459
Eicosenoicacid, C20:1	1.063 ± 0.158 ^B^	1.380 ± 0.125 ^AB^	1.700 ± 0.227 ^A^	0.012
Erucic acid, C22:1	0.028 ± 0.007 ^C^	0.638 ± 0.056 ^B^	0.821 ± 0.030 ^A^	<0.001
Nervonic acid, C24:1	0.014 ± 0.001 ^C^	0.044 ± 0.004 ^A^	0.035 ± 0.004 ^B^	<0.001
Polyunsaturated	6.815 ± 0.202	6.722 ± 0.250	7.095 ± 0.305	0.257
Linoleic acid, C18:2	1.826 ± 0.095 ^B^	1.652 ± 0.072 ^B^	2.575 ± 0.117 ^A^	<0.001
Linolenic acid, C18:3	0.016 ± 0.004 ^B^	0.021 ± 0.003 ^AB^	0.026 ± 0.002 ^A^	0.030
Eicosadienoic acid, C20:2	0.356 ± 0.040 ^B^	0.448 ± 0.057 ^A^	0.386 ± 0.010 ^AB^	0.079
Eicosatrienoic acid, C20:3	0.091 ± 0.010 ^A^	0.077 ± 0.006 ^A^	0.053 ± 0.005 ^B^	0.002
Arachidonic acid, C20:4	0.465 ± 0.043	0.446 ± 0.021	0.421 ± 0.012	0.232
Eicosapentaenoic acid, C20:5	3.725 ± 0.095 ^A^	3.405 ± 0.098 ^B^	3.059 ± 0.202 ^C^	0.004
Docosadienoic acid, C22:2	0.086 ± 0.010 ^C^	0.182 ± 0.004 ^A^	0.144 ± 0.005 ^B^	<0.001
Docosatetraenoic acid, C22:4	0.075 ± 0.006 ^B^	0.097 ± 0.007 ^A^	0.079 ± 0.004 ^B^	0.007
Docosapentaenoic acid, C22:5	0.034 ± 0.011	0.034 ± 0.006	0.028 ± 0.004	0.592
Osbond acid, C22:5	0.011 ± 0.001 ^A^	0.009 ± 0.001 ^B^	0.009 ± 0.000 ^B^	0.014
Docosahexaenoic acid, C22:6	0.129 ± 0.018 ^B^	0.350 ± 0.020 ^A^	0.314 ± 0.065 ^A^	0.001
Unsaturated/saturated ratio	0.239 ± 0.017 ^B^	0.315 ± 0.023 ^A^	0.298 ± 0.013 ^A^	0.004

* Different letters within each row indicate significant difference (*p* < 0.05; *n* = 3).

## Data Availability

The data presented in this study are available from the corresponding author on reasonable request.

## Supplementary Materials

The following supporting information can be downloaded at: https://www.mdpi.com/article/10.3390/antiox13091085/s1, Figure S1: Classification of sperm alkaline comet assay gel electrophoresis in dwarf surf clam, magnification ×400. Grade 0: no damage, normal cell, tail length < 5%, nucleus was intact; Grade I: slightly damaged, tail length 5 to 20%; Grade II: moderately damaged, tail length 20–40%, obvious tail observed; Grade III: heavily damaged, tail length 40–95%, nucleus reduced significantly; Grade IV: totally damaged, tail length >95%, nucleus becomes dim or disappears altogether.
