# Supplementary material for: Coenzyme Q10 Improves the Post-Thaw Sperm Quality in Dwarf Surfclam Mulinia lateralis
Source: Antioxidants (Basel). 2024 Sep 4;13(9):1085. doi: 10.3390/antiox13091085 (PMC11429170; doi:10.3390/antiox13091085)
Supplement: Supplementary file 1 [file antioxidants-13-01085-s001.zip › antioxidants-3074841-supplementary.pdf]

Coenzyme Q10 improves the post-thaw sperm quality in dwarf surfclam *Mulinia lateralis*

Zhen Xu [1], Zujing Yang [1], Lisui Bao [2], Bei Lu [1,3], Xiaoxu Li [4], Xin Zhan [5], Xiaoting Huang [1,*] and Yibing Liu [6,*]

1 Key Laboratory of Marine Genetics and Breeding (Ministry of Education), College of Marine Life Sciences, Ocean University of China, Qingdao 266003, China;
2 Institute of Evolution & Marine Biodiversity, Ocean University of China, Qingdao 266003, China;
3 Fang Zongxi Center for Marine EvoDevo, Ocean University of China, Qingdao 266100, China
4 Aquatic Sciences Centre, South Australian Research and Development Institute, Adelaide 5024, Australia;
5 School of Marine Biology and Fisheries, Hainan University, Haikou 570228, China;
6 Key Laboratory of Mariculture (Ministry of Education), Fisheries College, Ocean University of China, Qingdao 266003, China
* Correspondence: xthuang@ouc.edu.cn (X.H.); liuyibing@ouc.edu.cn (Y.L.)

Supplementary Figure

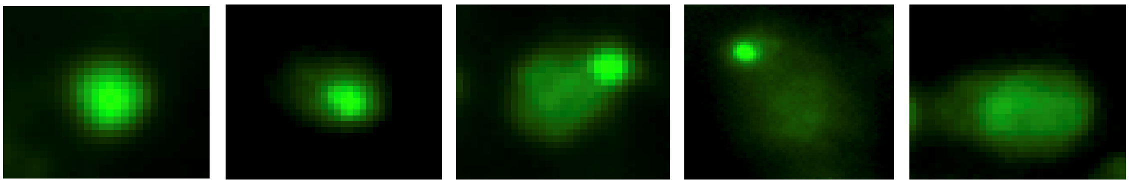

Grade 0 Grade I Grade II Grade III Grade IV

Figure S1. Classification of sperm alkaline comet assay gel electrophoresis in dwarf surf clam, magnification × 400. Grade 0: no damage, normal cell, tail length < 5%, nucleus was intact; Grade I: slightly damaged, tail length 5 to 20%; Grade II: moderately damaged, tail length 20-40%, obvious tail observed; Grade III: heavily damaged, tail length 40-95%, nucleus reduced significantly; Grade IV: totally damaged, tail length >95%, nucleus becomes dim or disappears altogether.
